# Acoustic Doppler velocimetry (ADV) data on flow-vegetation interaction with natural-like and rigid model plants in hydraulic flumes

**DOI:** 10.1016/j.dib.2020.106080

**Published:** 2020-07-25

**Authors:** Gerardo Caroppi, Kaisa Västilä, Paola Gualtieri, Juha Järvelä, Maurizio Giugni, Paweł M. Rowiński

**Affiliations:** 1 Department of Civil, Architectural and Environmental Engineering, University of Naples Federico II, Naples, Italy; 2 Department of Built Environment, Aalto University School of Engineering, Espoo, Finland; 3 Freshwater Centre, Finnish Environment Institute, Helsinki, Finland; 4 Institute of Geophysics, Polish Academy of Sciences, Warsaw, Poland

**Keywords:** Flow velocity, Acoustic Doppler velocimetry, ADV, Vegetation, Reconfiguration, Turbulence, Open channel flows

## Abstract

Vegetation, generally present along river margins and floodplains, governs key hydrodynamic processes in riverine systems. Despite the flow-influencing mechanisms exhibited by natural vegetation and driven by its complex morphology and flexibility, vegetation has been conventionally simulated by using rigid cylinders. This article presents a dataset obtained from hydraulic experiments performed for investigating the flow-vegetation interaction in partly vegetated channels. Vegetation was simulated by using both natural-like and rigid model plants. Specifically, two sets of experiments are described: in the first, vegetation was simulated with natural-like flexible foliated plants standing on a grassy bed; in the second, rigid cylinders were used. Experiments with rigid cylinders were designed to be compared against tests with natural-like plants, as to explore the effects of vegetation representation. The following experimental data were produced: 3D instantaneous velocity measured by acoustic Doppler velocimetry, vegetation motion video recordings, and auxiliary data including detailed vegetation characterization. These experiments are unique both for the use of natural-like flexible woody vegetation in hydraulic experiments and for the similarity achieved between the resulting observed vegetated shear layers. These data are expected to be useful in vegetated flows model development and validation, and represent a unique benchmark for the interpretation of the flow-vegetation interaction in partly vegetated channels.

**Specifications Table**

**Table:** 

**Subject**	Water Science and Technology
**Specific subject area**	Ecohydraulics, Open channel flows
**Type of data**	ADV Data, Excel File, Text, Table, Figure, Video
**How data were acquired**	Acoustic Doppler velocimetry (ADV) technique in laboratory controlled conditions
**Data format**	Raw and analyzed
**Parameters for data collection**	Velocity measurement carried out for steady, fully developed turbulent flows in partly vegetated channels.
**Description of data collection**	3D instantaneous point velocity measured with ADV at 200 and 100 Hz, for duration of 120 and 240 s, respectively. Velocity was investigated along mid-depth transects in uniform flow conditions.
**Data source location**	Environmental Hydraulics Lab, Aalto University, Espoo, Finland Laboratory of Hydraulics, University of Naples Federico II, Naples, Italy
**Data accessibility**	Repository name: Mendeley Data Data identification number: 10.17632/p6m666p4kb.1 Direct URL to data: https://data.mendeley.com/datasets/p6m666p4kb/1

**Value of the data  **

• These data were obtained with reference to two vegetation representations in partly vegetated channels: a novel experimental arrangement in which vegetation was simulated by using artificial flexible foliated plant stands and a second configuration with rigid cylinders. The value of these data relies in the hydraulic similarity achieved between the two sets of experiments and in the use of natural-like vegetation of complex morphology.
• River engineers, environmental scientists and researchers interested in flow-vegetation interaction and hydrodynamic processes in partly vegetated channels can benefit from these data.
• The described data are optimal for investigating the flow-vegetation interaction in partly vegetated channels, with reference to the mean and turbulent flow structure. These data can be used for developing and validating models for velocity prediction for vegetated flows.

## 1 Data description

The data described in this paper refer to two sets of experiments indicated as F and R, respectively. The laboratory setup, the experimental conditions, the measurement technique and the test cases are described in detail in the following Section 2 for both the sets of experiments.

For each experimental set three conditions are described, indicated as F1, F2 and F3, for F experiments, and R1, R2 and R3 for R experiments. The following experimental data were produced: 3D instantaneous point velocity measured by acoustic Doppler velocimetry (ADV), natural-like vegetation motion video recordings, flow discharge and free surface elevation. ADV raw and analyzed data, and vegetation video recordings are available at Mendeley Data [1]. The data in the archive are organized as schematized in Table 1. For F tests, video files (.mov) of the experiments are included into the archive. Data on vegetation characterization, flowrate and water depth are reported in this article. These data were collected in the framework of a larger experimental investigation on flow-vegetation interaction in partly vegetated channels [2,3].

**Table 1:** Data archive structure

Experiment set sub-folder	Test case sub-folder	Sub-folder	Sub-folder	Sub-folders
F_Data	F1	F1_ADV_Data	F1_Transect_1	ADV_Raw; ADV_Post-processed
			F1_Transect_2	ADV_Raw; ADV_Post-processed
		F1_Videos		
	F2	F2_ADV_Data	F2_Transect_1	ADV_Raw; ADV_Post-processed
			F2_Transect_2	ADV_Raw; ADV_Post-processed
		F2_Videos		
	F3	F3_ADV_Data	F3_Transect_1	ADV_Raw; ADV_Post-processed
			F3_Transect_2	ADV_Raw; ADV_Post-processed
		F3_Videos		
R_Data	R1	R1_ADV_Data	-	ADV_Raw; ADV_Post-processed
	R2	R2_ADV_Data	-	ADV_Raw; ADV_Post-processed
	R3	R3_ADV_Data	-	ADV_Raw; ADV_Post-processed

**Fig. 1:**
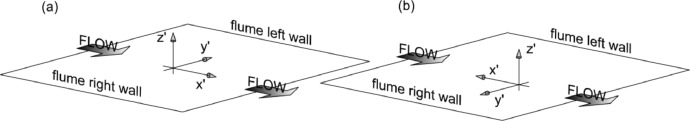
ADV probe reference system for down-looking and left-looking probe (a), and for right-looking probe (b).

**Fig. 2:**
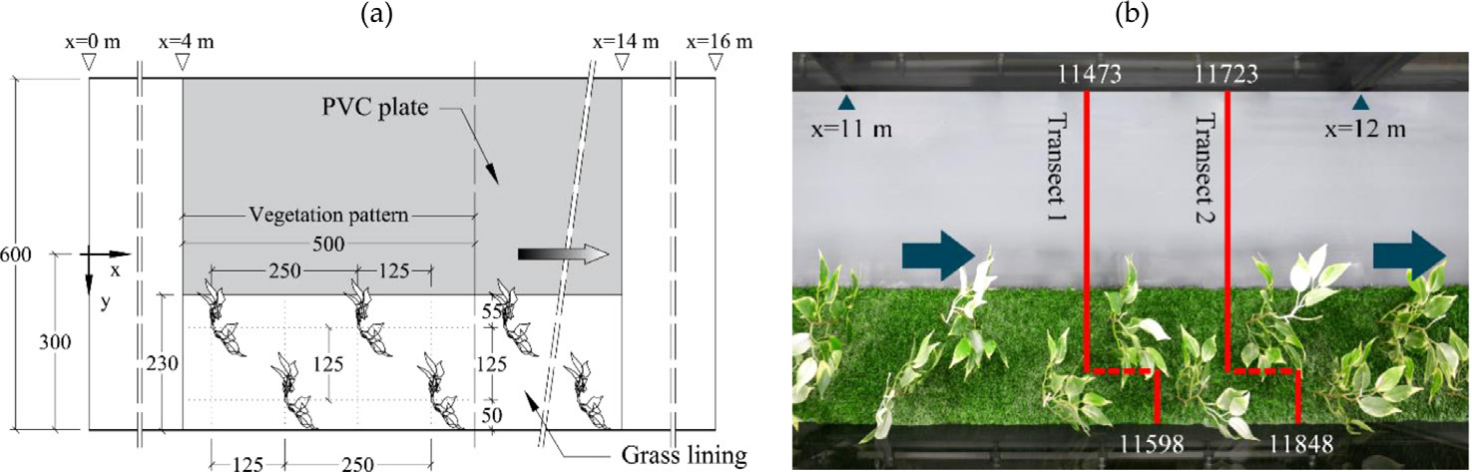
Top view of the F experimental facility with specification of coordinate system (a) and picture of the experimental flume with position of the two investigated transects (b) (modified from [2]).

Criteria used for naming the ADV raw point data files are specified in the following Section 2. For each raw ADV point measurement three files are provided: (1) the instrument original output file (.vno and .ntk for F and R experiments, respectively) with velocity time-series and probe setting information, and (2) two text files, one .dat file containing the velocity time-series and one .hdr file with the information on probe settings. The instrument output files can be read using dedicated software, the text files contain the same information and are provided for completeness. ADV data velocity components *u, v* and *w* are referred to the *x*’, *y*’ and *z*’ directions of the probe right-handed coordinate system. For all the measurements, the probe *x*’ axis was aligned to the mean flow direction, thus with the flumes *x* axis (Fig. 2 and Fig. 6). The correspondence between the other directions in the two reference systems can be deduced from the probe type (down- or side-looking) and its orientation, as schematized in Fig. 1. Right-looking (RL) and left-looking (LL) probe indicate a side-looking probe oriented towards the flume right and left wall, respectively. ADV data quality indicators, such as signal to noise ratio and signal correlation, are embedded into the output files.

### 1.1 ADV post-processed data

Before analysis, ADV data should be filtered from spikes and poor quality data [4], [5], [6]. In addition, filtered out values should be replaced to have a complete time-series [6]. Therefore, along with the raw ADV measurements, post-processed data, filtered and despiked using the Velocity Signal Analyser software (v1.5.64) [7], were included in this dataset. Specifically, values with signal to noise ratio and correlation lower than 15 dB and 70%, respectively, were filtered out. Spikes were detected using the modified phase-space thresholding method [5] with *C*_1_ and *C*_2_ equal to 1.48 [5,8], adopting the standard deviation as characteristic scalar. Filtered out values were replaced with the last good (valid) value in the time-series [6,7]. The average percentage of good data for all the ADV measurements included in this dataset is greater than 90%.

Post-processed ADV data are included into the dataset as Matlab data file (.mat). For each transect, in the ADV_Post-processed folder, two files are provided: uvw.mat and y.mat. The first file is a 24000 × 3x*N* numeric matrix containing, for each of the *N* measurement points, a 24000 element array with the filtered time-series for the three velocity components (in the order *u, v* and *w*). The y.m file is a *N* elements numeric array, containing the *y* coordinate of the measurement points. Post-processed velocities are provided so that *u* is positive along the flume *x* axis, *v* is negative along the flume *y* axis and *w* is positive along flume *z* axis.

### 1.2 Test summary file

In the main data folder, the key velocity statistics are provided in the SUM.xslx file. For each point measurement, the mean velocities and the elements of the velocity covariance matrix are provided. The reported statistics are evaluated with reference to the post-processed ADV data.

## 2 Experimental design, materials and methods

In the following Sections 2.1 and 2.2, the laboratory setup, the experimental conditions, the measurement technique and the test cases are described for F and R experiments, respectively.

### 2.1 F experiments

#### 2.1.1 Setup and vegetation representation

Experiments were carried out at the Environmental Hydraulics Lab of Aalto University, in a 20 m long, 0.6 m wide, and 0.8 m deep tilting glass-walled recirculating flume. In a 10 m long reach, starting from *x*=4 m, the flume bed was covered with a 20 mm thick PVC base plate that spanned the channel width, in which 2.5 mm diameter holes were drilled to install plant stands. The *x, y*, and *z* axes of the coordinate system refer to the longitudinal, lateral, and vertical (normal to the flume bottom) directions, respectively (Fig. 2, a). The coordinate system origin was defined as *x*=0 at the flume inlet, positive downstream; *y*=0 at the channel midline and positive towards the vegetation and *z*=0 at the top of the PVC bed plate and positive upwards.

**Fig. 3:**
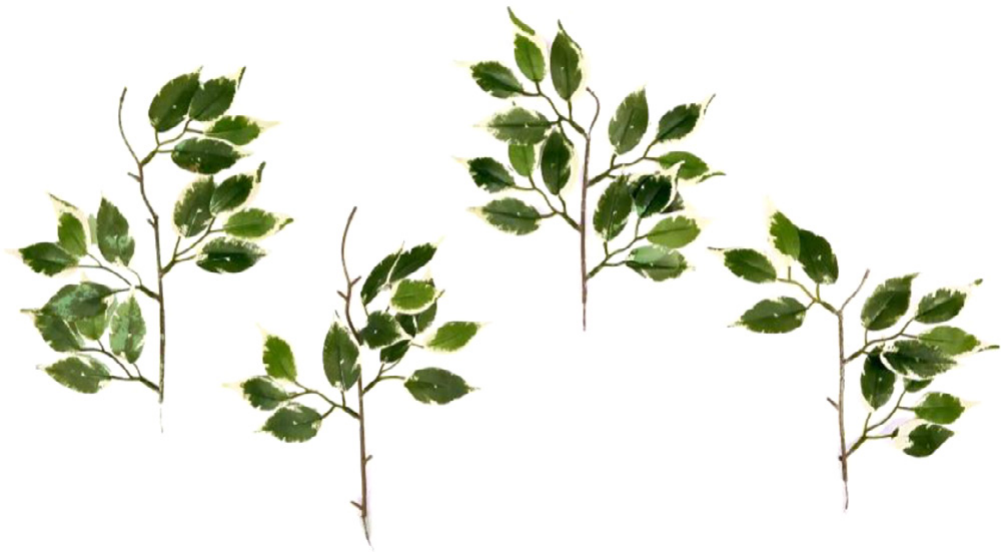
Picture of the 4 plant stands composing the repetitive pattern.

**Fig. 4:**
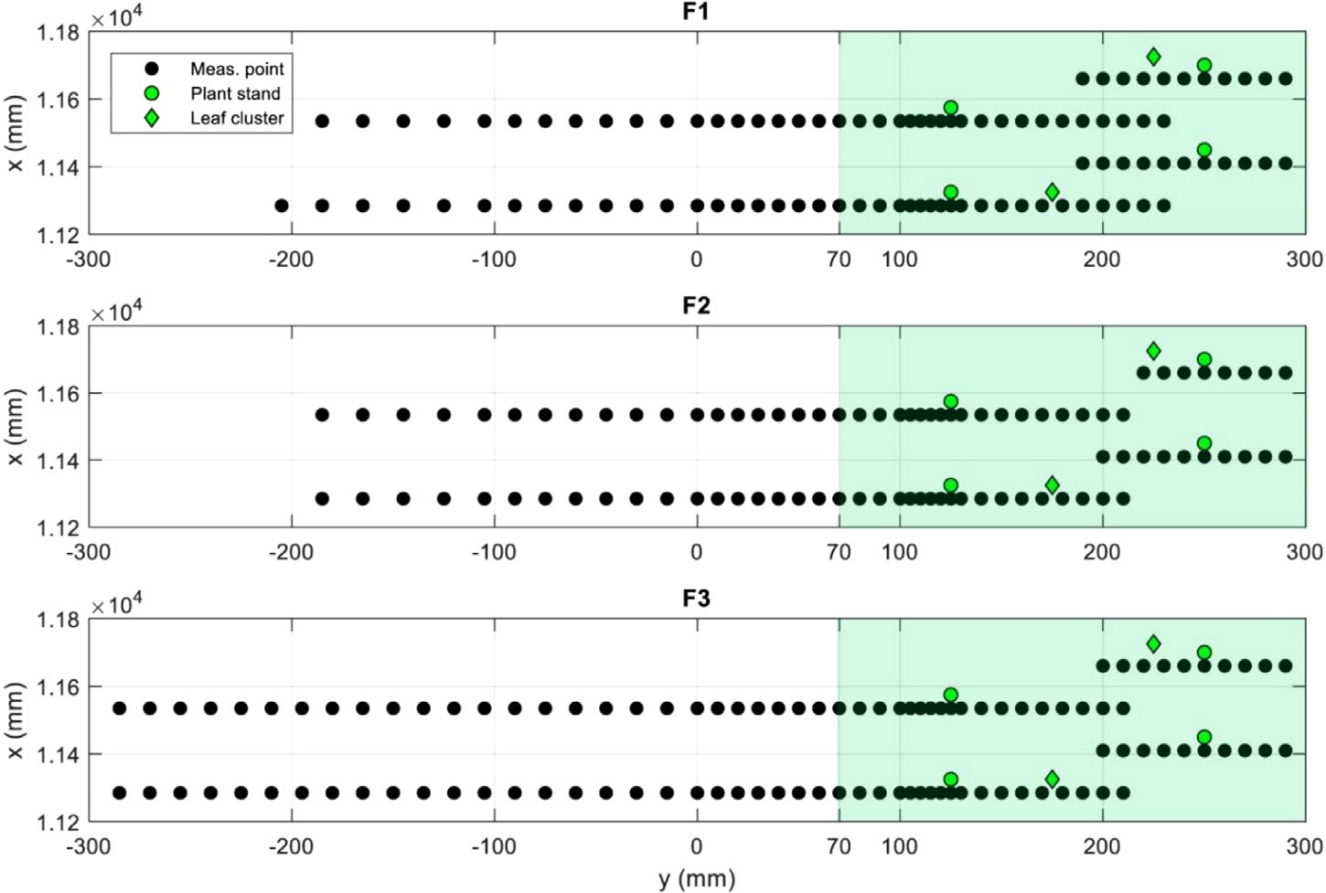
Coordinates (***x*** and ***y***) of measurement points for F tests with specification of vegetation elements position and grasses (light green hatched area).

Natural vegetation was simulated using artificial plants, with their properties carefully tested to reproduce similar behavior as natural floodplain vegetation. Different plant stands were built using modular plant elements: 205 and 270 mm tall stems with 3 mm average diameter, and leaf clusters. Stems were of polyethylene with an inner steel wire and presented short branches to which the leaf clusters were connected. Each leaf cluster was made of 4 polyester fabric leaves connected to short polyethylene petioles. The channel was vegetated using a repetitive pattern composed of 4 different plant stands (Fig. 3) and two lower leaf clusters, as to minimize preferential flow paths within the vegetated region. The position of the plants are specified in Fig. 2 and Fig. 4.

The plants were arranged in two staggered rows, with a spacing of 250 and 125 mm in the *x* and *y* directions, respectively (Fig. 2), resulting in a number of plant stands per m^2^ of vegetated area equal to 35. The frontal area per unit volume evaluated for stems and leaves within the vegetation pattern was equal to 0.13 and 4.1 m^−1^, respectively. A 20 mm thick and 230 mm wide artificial turfgrass (frontal area per canopy volume ≈500 m^−1^) was selected as bed roughness in the vegetated area of the flume.

The flow into the flume was driven and recirculated with a pumping system. From the inlet tank the water flowed to the flume through a flow straightener dampening inlet related turbulence and surface instabilities. The discharge was measured at 80 Hz with a magnetic flowmeter (accuracy 0.5%). 6 pressure transducers tapped along the flume bed midline (at *x*=5, 6.5, 8, 9.5, 11.5 and 13 m) were used to measure the water depth in the vegetated reach of the flume (accuracy ± 3 mm). Uniform flow conditions were attained by regulating the flume bed slope and the position of a downstream tailgate. For all the test runs, the submerged leaf area was approximately constant, being the plants just submerged.

#### 2.1.2 Test runs and velocity measurements

Three conditions, indicated as F1, F2 and F3 were tested. The flow rate *Q*, the water depth *h*, the cross-section averaged velocity *U_m_*, and the flume slope *i* are specified in Table 2.

**Table 2:** Test cases for F experiments

Test Run	*Q* (l/s)	*h* (mm)	*U_m_* (m/s)	*i* (%)
F1	22.2	170	0.22	0.107
F2	50.0	170	0.49	0.336
F3	83.0	170	0.82	0.707

#### Velocity measurements and data records

Instantaneous three-dimensional velocity components were measured at 200 Hz with Nortek Vectrino+ ADV with a 4-beam side looking probe, to the accuracy of ± 1%. The cylindrical sampling volume was 50 mm far from the probe tip and was equal to ≈198 mm^3^ (7 mm long with 6 mm diameter). ADV velocity range and probe settings information are included in point measurement files. In order to achieve and maintain good signal-to-noise ratio and signal correlation during the tests, the flow was seeded with 7–10 μm diameter solid glass micro-spheres. A recording time of 120 s (24000 samples) allowed to achieve constant higher order velocity moments and was used for each point measurement.

Velocity measurements were carried along two lateral mid-depth transects, indicated as Transect 1 and Transect 2 (Fig. 2, b). Specifically, the transects vertical coordinate (*z*=95 mm) was defined taking into account the 20 mm tall bed grasses, as to correspond to *h*/2 in the vegetated region. Each transect was composed of two segments: one located deeper into the vegetated region near the flume right wall, spanning from *y*=300 mm to *y*=200 mm, and another shifted 125 mm upstream and covering the rest of the cross-section. The distance between the investigated transects and the upstream and downstream closest plants was equal to 40 and 210 mm, respectively. The details of the measuring points position within the flow domain and the position of vegetation elements at the bed are indicated in Fig. 4. For each transect, velocity measurements were taken every 5–20 mm, with the lowest spacing used in the region with the highest flow lateral variability. Measurement and transect locations were defined as to have the flow domain accessible by ADV probe for all the test cases, with no plant elements entering the path of acoustic beams.

Each point velocity time-series ADV raw file is named as *y*-*z;x*_A_B.vno, with *y, z* and *x*, in mm, indicating the lateral, vertical and longitudinal coordinate in the flume reference system; A indicates the probe orientation (being equal to RL or LL for right- and left-looking probe, respectively); B indicates the ADV used for the tests. The comment B was added for the sake of completeness but only one instrument, indicated with W2, was used. Thus, starting from F2 test case, this comment was omitted. The *x* coordinate used for naming the ADV data is shifted of 188 mm upstream of the actual *x* value. With reference to Fig. 2, data for transect 11598–11473 are indicated with *x* coordinate equal to 11410 mm and 11285 mm, while data for transect 11848–11723, are indicated with *x* coordinate equal to 11660 mm and 11535 mm, as indicated in Fig. 4.

#### Video-records of F experiments

**Fig. 5:**
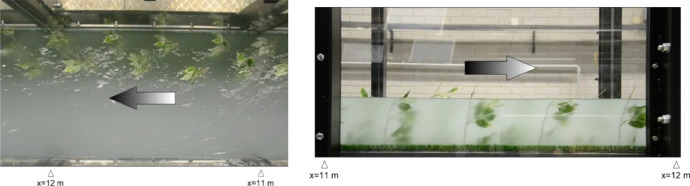
Frame for top (a) and side (b) video (F2 test case) with specification of longitudinal location and flow direction.

For each test case, two videos at 25 fps with duration >90 s were taken with a Nikon D5100 Camera to track the vegetation motion during the tests. Videos were taken from the top and the hydraulic right-handed side of the flume, as an example, two cropped frames with specification of the longitudinal location and referred to test case F2 are reported in Fig. 5. For top videos the water flows from right to left, for side videos water flows from left to right.

### 2.2 R experiments

#### 2.2.1 Setup and vegetation representation

**Fig. 6:**
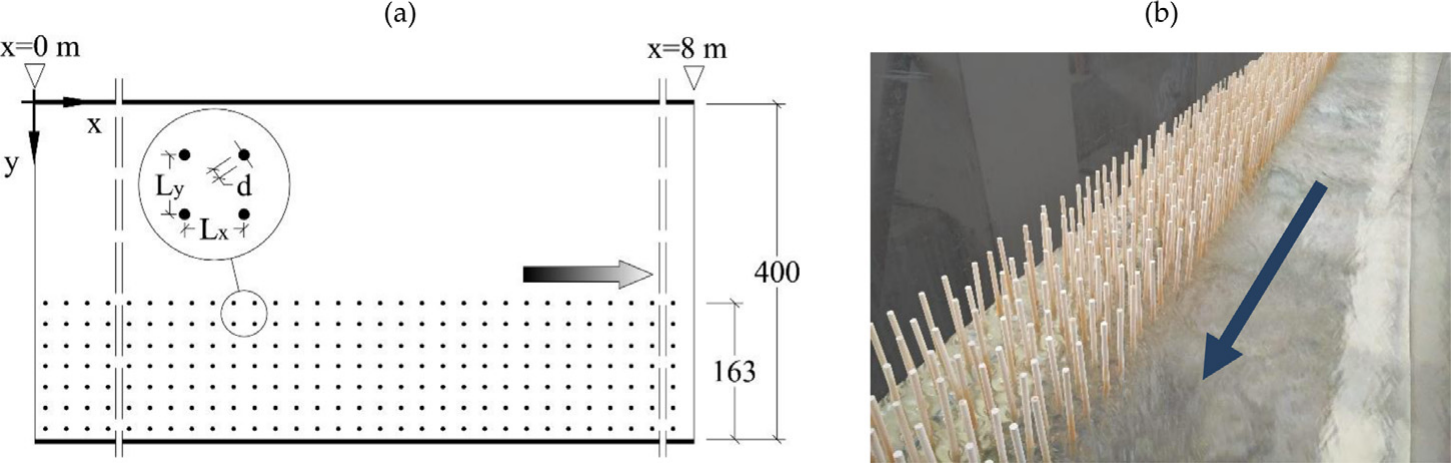
Top view of the R experimental facility (a, modified from [3]) and picture of the flume with emergent cylinders (b).

Experiments were carried out at the Laboratory of Hydraulics of University of Naples Federico II, in a 8 m long and 0.4 m wide flume with 0.48% fixed slope. The flume bed was covered with acrylic panels presenting a grid of holes with diameter equal to 4 mm for cylinders insertion. The flume coordinate system was defined setting *x*=0 at the inlet cross-section, positive downstream; *y*=0 at the channel hydraulic left wall positive towards the channel and *z*=0 at the top of the acrylic bed plate and positive upwards (Fig. 6).

Vegetation was simulated by using 190 mm tall rigid wooden cylinders with 4.5 mm diameter (the base tip was shaped as to fit the 4 mm holes). The flume bed was partly covered by a 163 mm wide array of aligned emergent cylinders (Fig. 6).

The flow entered the experimental flume from a tank equipped with a set of vanes as to dissipate waves. In order to reduce inlet-related disturbance, the flow entered the flume via a parabolic transition. A centrifugal pumping system placed in a tank downstream of the flume outlet recirculated the flow through the laboratory pipeline system. The discharge was measured with a magnetic flowmeter (accuracy 0.5%). The free surface elevation along the flume was manually measured with a gauging needle (with nominal accuracy of 0.1 mm).

#### 2.2.2 Test runs, velocity measurements and data records

Three cylinder densities were tested, resulting in three test cases, namely R1, R2 and R3. The flow rate *Q*, the water depth *h* in the measuring reach, the cross-section averaged velocity *U_m_*, the cylinder spacing *L_x_* and *L_y_*, in the *x* and y direction, respectively, and the longitudinal position of the investigated transect are specified in Table 3.

**Table 3:** Test runs and cylinder arrangement specification for R tests.

Test Run	*Q* (l/s)	*h* (mm)	*U_m_* (m/s)	*L_x_* (mm)	*L_y_* (mm)	Transect longitudinal location (m)
R1	15.2	84	0.45	25	25	∼3.4 (section 44)
R2	31.7	135	0.59	25	50	∼3.6 (section 42)
R3	31.7	122	0.65	100	50	∼3.7 (section 41)

**Fig. 7:**
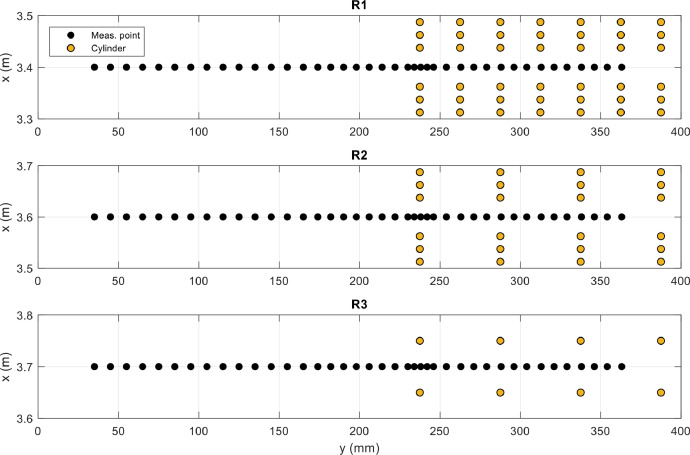
Coordinates (***x*** and ***y***) of measurement points for R test cases with specification of cylinder position.

Instantaneous three dimensional velocity components were measured with acoustic Doppler velocimetry at 100 Hz with a Nortek Vectrino II ADV with a 4-beam down looking probe, to the accuracy of ± 1%. The sampling volume was 40 mm far from the probe tip with a volume of ≈154 mm^3^ (4 mm long with 7 mm diameter). ADV velocity range and probe settings information are included in point measurement files. The scatter material present into the water was sufficient for achieving and maintaining good signal-to-noise ratio for the entire duration of the tests. A recording time of 240 s (24000 samples) allowed to achieve constant higher order velocity moments and was used for each point measurement. To allow the probe access within the cylinder array, for test case R1 and R2 two rows of cylinders were removed (Fig. 7).

For each test case velocity measurements were carried out along one mid-depth (*z*=*h*/2) transect. The transect longitudinal locations specified in Table 3 correspond to section 44, section 42 and section 41 of the experimental flume, for R1, R2 and R3, respectively. The detail of the measuring points position within the flow domain and the relative position of the cylinders is indicated in Fig. 7. Velocity measurements were taken every 4-10 mm, with the lowest spacing used in the region with the highest flow lateral variability.

Each point velocity time-series ADV raw file is named as *y*-*z*;S.A.ntk, with *y, z* indicating the lateral and vertical coordinate of the measuring point, in mm, in the flume reference system; S indicates the flume longitudinal section of the transect (the correspondence between section number and longitudinal position of the transect is indicated in Table 3). A indicates an alphanumerical string automatically added to the filename by the instrument software, its meaning has no relevance to the interpretation of the dataset described herein.

## Declaration of Competing Interest

The authors declare that they have no known competing financial interests or personal relationships which have, or could be perceived to have, influenced the work reported in this article.

## Appendix Supplementary materials

Image, application 1
